# Supplementary material for: Empirical methods for controlling false positives and estimating confidence in ChIP-Seq peaks
Source: BMC Bioinformatics. 2008 Dec 5;9:523. doi: 10.1186/1471-2105-9-523 (PMC2628906; doi:10.1186/1471-2105-9-523)
Supplement: Additional File 1 — A variety of html documents from the USeq web site detailing the available applications, their best usage, output file type descriptions, command line menus, etc. [file 1471-2105-9-523-S1.zip › USeqUserGuides/JavaDoc.zip!/JavaDoc/trans/main/class-use/Oligo.html]

Uses of Class trans.main.Oligo


|  |  |  |
| --- | --- | --- |
| **Overview** \| **Package** \| **Class** \| **Use** \| **Tree** \| **Deprecated** \| **Index** \| **Help** | |  |
| PREV   NEXT | **FRAMES**    **NO FRAMES**     **All Classes** |  |


---


## **Uses of Class trans.main.Oligo**

| Packages that use Oligo | |
| --- | --- |
| **trans.graphics** | For graphically representing '.cel' files and intervals. |
| **trans.main** | Principle classes and scripts used in TiMAT2 analysis. |

Uses of Oligo in trans.graphics

| Methods in trans.graphics with parameters of type Oligo | |
| --- | --- |
| `static double` | `IntervalDrawPanel.fetchClosestOligoScore(Oligo[] oligos, int position)`             Finds the closes Oligo to a bp point and returns the indexed score. |
| `static double` | `IntervalDrawPanel.fetchMaxOligoScore(Oligo[] oligos, int startBp, int stopBp)`             Scans oligos for a max score when given a bp range. |

Uses of Oligo in trans.main

| Methods in trans.main that return Oligo | |
| --- | --- |
| `Oligo[]` | `Interval.extractSubRegionOligos(int start, int end)`             Given a bp start and bp end as well as a larger Oligo[] returns a sub array of the oligos flanking and including the start and end. |
| `Oligo[]` | `SubWindow.getOligos()` |
| `Oligo[]` | `PeakPicker.getOligos()` |
| `Oligo[]` | `Interval.getOligos()` |

| Methods in trans.main with parameters of type Oligo | |
| --- | --- |
| `static double` | `IntervalReportPrinter.calculateMeanNumberOligoMatches(Oligo[] oligos)`             Calculates the average number of oligo matches to the genome. |
| `static void` | `IntervalGFFPrinter.gff(Oligo o, Interval i, java.lang.String name, int oligoNumber, java.lang.StringBuffer s)`             Appends a GFF3 line for an Oligo. |
| `void` | `SubWindow.setOligos(Oligo[] oligos)` |
| `void` | `PeakPicker.setOligos(Oligo[] oligos)` |
| `void` | `Interval.setOligos(Oligo[] oligos)` |
| `java.lang.String` | `BindingPeak.toString(Oligo[] oligos, int oligoLength)`             Score, Left, Peak, Right |

---


|  |  |  |
| --- | --- | --- |
| **Overview** \| **Package** \| **Class** \| **Use** \| **Tree** \| **Deprecated** \| **Index** \| **Help** | |  |
| PREV   NEXT | **FRAMES**    **NO FRAMES**     **All Classes** |  |


---
